# Effect of Selenium-Enriched Donkey Milk on Lipid Metabolism Disorders Induced by a High-Fat Diet

**DOI:** 10.3390/foods15101640

**Published:** 2026-05-08

**Authors:** Qizhen Zhong, Jie Cheng, Gengli Huang, Julei Zhan, Xue Chen, Meixuan Liu, Guangyuan Liu, Zhuoru Ren, Xuemei Chen, Xiaoshu Tang, Zhouping Wang

**Affiliations:** 1 State Key Laboratory of Food Science and Resources, Jiangnan University, Lihu Road 1800, Wuxi 214122, China; 6230112176@stu.jiangnan.edu.cn (Q.Z.); 7220112082@stu.jiangnan.edu.cn (G.H.); 1011190127@stu.jiangnan.edu.cn (J.Z.);; 2 Shandong Key Laboratory of Gelatine Medicines Research and Development, Dong’e Ejiao Co., Ltd., Liaocheng 252200, China; chengjie@dongeejiao.com (J.C.);; 3 National Engineering Technology Research Center for Gelatin-Based Traditional Chinese Medicine, Dong’e Ejiao Co., Ltd., Liaocheng 252200, China; 4 National Engineering Research Center for Functional Food, Jiangnan University, Wuxi 214122, China; 5 Key Laboratory of Screening, Prevention, and Control of Food Safety Risks, State Administration for Market Regulation, Wuxi 214122, China; 6 School of Food Science and Technology, Jiangnan University, Wuxi 214122, China

**Keywords:** selenium-enriched donkey milk, short-chain fatty acid, Akkermansia muciniphila, AMP-activated protein kinase

## Abstract

Selenium-enriched donkey milk (Se-DM) is characterized by low fat levels, low casein levels, high antioxidant activity, and high selenium content. To date, the regulatory role of Se-DM in the context of a high-fat diet (HFD) is unclear, prompting this present study to elucidate the potential mechanisms by which it affects the gut microbiota and hepatic lipid metabolism in rats fed a high-fat diet. The effects of Se-DM on fat accumulation and lipid peroxidation in HFD rats were investigated through non-targeted fecal metabolomics, short-chain fatty acid (SCFA) quantification, 16S microbial analysis, and pathological assessments. The results showed that Se-DM enhanced SCFA production by promoting Akkermansia muciniphila (*A. muciniphila*) proliferation, thereby regulating the AMP-activated protein kinase pathway and ferroptosis. Immunoblotting of hepatic tissues and qPCR analyses confirmed the engagement of the AMP-activated protein kinase cascade, suppression of ferroptosis, and reduction in fatty acid synthesis-related proteins (FASN, CD36, SCD1). Our findings revealed that Se-enriched donkey milk (Se-DM) ameliorated lipid metabolism in rats, providing theoretical support for developing functional foods to treat hyperlipidemia.

## 1. Introduction

Lipid metabolism disorders involve abnormalities in fatty acid, triglyceride, and cholesterol metabolism and are influenced by both environmental and genetic factors. In recent years, changes in dietary patterns and lifestyles, particularly the prevalence of high-fat diets (HFDs), have been the major contributors to lipid metabolism disorders. The current strategies to mitigate lipid metabolism disorders mainly focus on inhibiting endogenous lipid synthesis, eliminating lipid peroxidation, promoting exogenous lipid metabolism, and regulating gut microbiota [1,2]. Statins remain the primary pharmacological intervention for HFD-related diseases. However, their side effects and potential treatment intolerance have led to increasing exploration of alternative therapies [3]. Functional foods recognized for their antioxidant, anti-inflammatory, cholesterol-lowering, and gut microbiota-regulating benefits, thereby helping to manage lipid metabolism disorders safely and with few side effects [4,5]. Therefore, developing functional foods with lipid metabolism-regulating potential is a promising strategy for preventing and managing obesity and related metabolic diseases.

Donkey milk features low fat, low casein, high lysozyme, and high selenium (Se) content; and it possesses multiple health-promoting properties, such as antioxidant, anti-tumor, anti-inflammatory, and hypoallergenic activities. These attributes render donkey milk as a recognized functional health food [6,7]. Studies have shown that dietary supplementation with Se-enriched yeast can reduce oxidative stress in animals, enhance milk yield during lactation, and increase protein and Se content in milk [8,9,10]. Se-fortified foods, with higher organic Se content, can significantly regulate colitis, obesity, oxidative damage, immune function, and gut microbiota dysbiosis [11,12]. However, the enhanced functional components in Se-DM and their potential impacts on gut homeostasis and lipid metabolism remain underexplored.

AMP-activated protein kinase (AMPK), a central sensor of cellular energy, maintains metabolic homeostasis by boosting glucose uptake and fatty acid oxidation, thereby regulating overall energy expenditure [13,14]. Studies have shown that AMPK activation ameliorates lipid metabolism disorders by reducing fat accumulation and lipid peroxidation. Gut microbial metabolites may be transported to the liver across the gut–liver axis, thereby activating the AMPK pathway [15,16]. Therefore, the present work examined whether Se-DM ameliorates HFD-induced hepatic lipid dysregulation by analyzing gut microbiota composition and metabolites. The results showed that Se-DM activated AMPK by modulating gut microbiota, mitigating HFD-induced ferroptosis, and reducing downstream SREBP1c-mediated lipid synthesis, thereby improving hepatic steatosis and inflammation.

Additionally, the effects of Se-DM on the intestinal microbiota and hepatic lipid homeostasis of rats maintained on a high-fat diet were examined. Inductively coupled plasma mass spectrometry (ICP–MS) was used to analyze the nutritional elements in Se-DM. The effects of Se-DM on fat accumulation and lipid metabolism disorders were examined using metabolomics and 16S rRNA sequencing. Notably, the study elucidates the mechanisms by which Se-DM enhances SCFA production through alterations in gut microbiota composition, thereby regulating lipid peroxidation and upstream signaling pathways of fatty acid synthesis. Additionally, functional components in Se-DM that promote probiotic growth were identified. These results support the development of functional dairy products supplemented with selenium to promote health.

## 2. Materials and Methods

### 2.1. Drugs and Chemicals

Donkey milk and Se-enriched donkey milk were supplied by Dong-E-E-Jiao Co., Ltd. (Jinan, Shandong, China). All chemicals were sourced from Solarbio (Beijing, China). The enzyme-linked immunoassay (ELISA) kits of adenosine monophosphate (AMP) and adenosine triphosphate (ATP) were obtained from Shanghai Meilian Industrial Co., Ltd. (Shanghai, China). The HFD containing 45% fat, 34% carbohydrate, and 20% protein (total energy content of 470 kcal/100 g) was sourced from Jiangsu Synergy Pharmaceutical and Bioengineering Co., Ltd. (Suzhou, China).

### 2.2. Determination of Nutritional Element Content and Amino Acid Composition

Samples of donkey milk and selenium-enriched donkey milk underwent defatting and freeze-drying after collection. ICP–MS was used to determine the contents of zinc (Zn), Se, calcium (Ca), and phosphorus (P). A Model L-8900 amino acid analyzer (Hitachi, Tokyo, Japan) was employed to quantify the 16 standard amino acids, following the Chinese National Standard protocol (GB 5009.124-2016) [17].

### 2.3. Determination of Organic Se Content

Samples of donkey milk and selenium-enriched donkey milk were collected, defatted, and lyophilized. The processed samples were refrigerated at −80 °C and then transported to Scientific Compass Co., Ltd. (Suzhou, China) for Se speciation analysis. The concentrations of various Se species, including selenite (Se^4+^), selenate (Se^6+^), selenomethionine (SeMet), selenocysteine (SeCys), and methylselenocysteine (MeSeCys), were quantitatively analyzed using HPLC-ICP-MS.

### 2.4. Animal Experiment

The experimental protocol was approved by the Animal Ethics Committee of Jiangnan University (Approval ID: JN. No 20250228S1100524[026]), and all animal procedures complied with the university’s guidelines for the Care and Use of Laboratory Animals. Specific pathogen-free male Sprague–Dawley (SD) rats (*n* = 40, 200 ± 10 g) were obtained from SpePharm Biotechnology Co., Ltd. (Suzhou, China) (License No. SCXK(Su)2022-0006). Rats were acclimated for one week under conditions of 24–26 °C, 50–60% humidity, and a 12 h light/dark cycle in an animal facility prior to the experiment. Rats were randomly divided into five groups (n = 8 per group) after acclimatization: normal chow (Control), HFD, donkey milk (DM), low-dose Se-DM (Se-L), and high-dose Se-DM (Se-H). The control group received a normal diet containing 10% fat-derived calories (XTCON50H), while the HFD, DM, Se-L, and Se-H groups were fed an HFD containing 45% fat-derived calories (XTHF45). The DM group was gavaged with 2.6 mL/kg/day of donkey milk, while the Se-L and Se-H groups were gavaged with 2.6 mL/kg/day and 10.4 mL/kg/day of Se-DM, respectively. Rats in the HFD and control groups were administered an equivalent volume of saline by gavage. After eight weeks of treatment, the rats were fasted for 12 h, after which they were placed in an airtight container and euthanized through the introduction of CO_2_ gas. Serum was isolated from abdominal aortic blood by centrifugation at 3500 rpm for 15 min at 4 °C. Liver, epididymal fat, and fecal samples were collected after euthanasia. Liver and epididymal fat were weighed and recorded. A portion of liver tissue and fecal samples were immediately refrigerated for later analysis after storage at −80 °C, while another portion of liver and epididymal fat tissues was fixed in 10% paraformaldehyde for histopathological examination.

### 2.5. Cell Culture and Treatment

HepG2 cells, a human hepatocellular carcinoma cell line, were obtained from Fenghui (Wuhan, Hubei, China). Cells were maintained in Dulbecco’s Modified Eagle’s Medium (Gibco, Waltham, MA, USA) containing 10% fetal bovine serum (New Cell & Molecular Biotech, Suzhou, China) and 1% penicillin/streptomycin (Beyotime, Shanghai, China) at 37 °C under a humidified atmosphere with 5% CO_2_. To examine the impact of SCFAs on ferroptosis in HepG2 cells, the cells were incubated for 24 h with 2.5 μmol/L erastin (MedChemExpress, Monmouth Junction, NJ, USA) in combination with either 1 mmol/L sodium acetate, 1 mmol/L sodium propionate, or 1 mmol/L sodium butyrate (Solarbio, Beijing, China). To assess the attenuation of ferroptosis by acetate in HepG2 cells via AMPK pathway activation, the cells were pretreated with or without 2 μmol/L Compound C (MedChemExpress, NJ, USA), an AMPK inhibitor, for 12 h, and then co-treated with 2.5 μmol/L of erastin and 1 mmol/L of sodium acetate for 24 h. For lipid peroxidation assessment, HepG2 cells were cultured at a seeding density of 1 × 10^4^ cells per mL into 35 mm glass-bottom confocal dishes (Biosharp Life Sciences, Beijing, China) and allowed to adhere overnight. Following the indicated treatments, cells were incubated with 2 μM C11-BODIPY 581/591 (Invitrogen, Thermo Fisher Scientific, Waltham, MA, USA), a ratiometric fluorescent probe sensitive to lipid peroxides, at 37 °C for 20 min in the dark.

After staining, the cells were gently washed three times with pre-warmed phosphate-buffered saline (PBS) to remove the unbound probe. Fluorescence imaging was then performed using a Zeiss LSM 880 confocal laser scanning microscope (Carl Zeiss AG, Oberkochen, Germany) equipped with appropriate excitation (488 nm) and emission filters to monitor the redox-dependent shift from red (~590 nm) to green (~510 nm) fluorescence, indicative of lipid peroxidation.

### 2.6. In Vitro Culture of Akkermansia Muciniphila

To explore how organic selenium components in Se-DM promote *A. muciniphila* growth, the strain was sourced from the National Microbial Culture Collection Center (ATCC, Suzhou, China). After *A. muciniphila* was revived, it was inoculated (5%, vol/vol) into test tubes filled with 10 mL of brain heart infusion medium (supplemented with 0.05% L-cysteine, Sangon Biotech, Shanghai, China). The medium was further supplemented with 2 mg/mL L-SeMet, L-SeCys, or lactose (Solarbio, Beijing, China). An anaerobic chamber was used to culture the samples at 37 °Cover a 60-h period.

### 2.7. Serum and Liver Biochemical Indicators

Serum levels of total triglycerides (TGs), total cholesterol (TC), high-density lipoprotein cholesterol (HDL-C), low-density lipoprotein cholesterol (LDL-C), aspartate aminotransferase (AST), alanine aminotransferase (ALT), and alkaline phosphatase (ALP) in rats were measured using a fully automated biochemical analyzer (Zybio, Beijing, China). We determined hepatic concentrations of triglycerides (TGs), total cholesterol (TC), malondialdehyde (MDA), superoxide dismutase (SOD), and ferrous ions using commercially available biochemical assay kits. Hepatic AMP and ATP levels were quantified with ELISA kits.

### 2.8. Histological Analysis

Portions of liver and epididymal adipose tissue were placed in 10% neutral buffered formalin for histology. Liver samples underwent hematoxylin and eosin (H&E) staining and Oil Red O staining, whereas epididymal fat was processed for Oil Red O staining. Morphological assessment and lipid deposition analysis were performed on the stained tissue sections using a light microscope (Leica Microsystems, Solms, Germany).

### 2.9. RNA Extraction and Quantitative Real-Time PCR

Total RNA from liver tissues was extracted with Trizol reagent (Takara, Tokyo, Japan). The resulting RNA was then reverse-transcribed into complementary DNA (cDNA) using the Mighty Script First Strand cDNA Synthesis Master Mix (Sangon Biotech, Shanghai, China). We conducted RT-qPCR on a LightCycler 480 II system (Roche, Basel, Switzerland) using 2X SG Fast qPCR Master Mix (High Rox) (Sangon Biotech, Shanghai, China). The mRNA levels of target genes (LKB1, ACSL4, Slc7a11, GPX4, Srebp1c, PPAR-γ) were normalized to GAPDH, and relative expression was calculated using the 2^−ΔΔCt^ method. Primer sequences are provided in Table S1.

### 2.10. Microbial Profile Analysis

The Soil Genomic DNA Extraction Kit (Solarbio, Beijing, China) was used for genomic DNA extraction from the samples as per the manufacturer’s instructions. Each PCR reaction contained 20 μL of PCR Master Mix (2X) (K0171/K0172) (Sangon Biotech, Shanghai, China). The TruSeq^®^ DNA PCR-Free Sample Preparation Kit (Illumina, San Diego, CA, USA) was used for library preparation according to the manufacturer’s specifications, with index sequences incorporated. Amplicon sequence variants (ASVs) were derived from sequencing reads, and subsequent analyses were carried out via the Metware cloud platform (https://cloud.metware.cn).

### 2.11. Metabolomics in Feces Based on UHPLC-Q-TOF MS Analysis

For fecal sample processing, specimens were stored at −80 °C until use. Extraction was performed by mixing 20 mg of each sample with 500 μL of methanol-water (9:1, *v*/*v*) spiked with an internal standard, followed by vortexing for 3 min. The mixture was then sonicated in an ice water bath for 15 min, vortexed for an additional 90 s, and left at −20 °C for 30 min. Following centrifugation at 11,000 rpm for 20 min at 4 °C, the resulting pellet was discarded and the supernatant retained. The supernatant was subjected to another round of centrifugation at 11,000 rpm for 3 min at 4 °C. From the resulting solution, 200 μL was taken for LC–MS analysis. Each sample was prepared in duplicate. Metabolites that were identified underwent annotation using the KEGG Compound database (http://www.kegg.jp/kegg/compound/ (accessed on 10 March 2025)). Subsequently, we assigned these annotated metabolites to relevant pathways using the KEGG pathway database (http://www.kegg.jp/kegg/pathway.html (accessed on 12 March 2025)).

### 2.12. Fecal SCFA Analysis

First, a 30 mg fecal specimen was transferred into an EP tube. Subsequently, 0.5 mL of phosphoric acid solution (1%, *v*/*v*) along with a small steel bead were introduced into the tube. After thorough homogenization, the mixture was vortexed for 10 min and then sonicated for 5 min. Next, from the upper aqueous layer, a 100-μL aliquot was taken and placed in a fresh 1.5 mL centrifuge tube. Centrifugation was then performed at 11,000 rpm for 10 min at 4 °C. Then, for internal standard calibration, 500 μL of Methyl tert-butyl ether (MTBE) spiked with the standard was added to the tube. The resulting mixture was vortexed for 3 min and sonicated for 5 min. Centrifugation was then repeated under identical conditions (11,000 rpm, 10 min, 4 °C), and the organic supernatant was retained for GC–MS/MS analysis.

Short-chain fatty acids were quantified on a GC-MS (Agilent 7890B, Santa Clara, CA, USA) fitted with a DB-FFAP capillary column (J&W Scientific, Santa Clara, CA, USA). Acquisition was performed in multiple reaction monitoring (MRM) mode. Temperature settings were as follows: injector port at 250 °C and transfer line at 230 °C.

### 2.13. Western Blot

Liver tissues or cellular proteins were homogenized on ice using RIPA lysis buffer (ACE Biotechnology, Changzhou, China) supplemented with protease and phosphatase inhibitors (NCM Biotech, Hangzhou, China). Protein quantification was performed with a bicinchoninic acid (BCA) assay kit (Solarbio, Beijing, China). Equal amounts of protein were resolved by SDS-PAGE and transferred onto PVDF membranes (Millipore Corp., Bedford, MA, USA). After blocking with 5% non-fat milk in PBST for 2 h at room temperature, the membranes were probed with primary antibodies against target proteins (FASN, SCD1, AMPKα, p-AMPKα, SirT1, PGC-1α, PPAR-α) and the internal control. Following washes, HRP-conjugated secondary antibodies were applied. Immunoreactive bands were detected using an enhanced chemiluminescence (ECL) kit. Signal intensities were quantified with ImageJ 1.8 software (National Institutes of Health, Bethesda, MD, USA), with β-actin used as the loading control. A complete list of antibody details is provided in Table S2.

### 2.14. Statistical Analysis

All values are expressed as mean ± standard error of the mean (SEM). Prior to analysis, the normality of data distribution was assessed using the Shapiro–Wilk test, and homogeneity of variances was evaluated using Levene’s test. All data met the assumptions of normality (*p* > 0.05) and homogeneity of variances (*p* > 0.05). Group comparisons were performed using one-way analysis of variance (ANOVA), followed by Tukey’s HSD multiple comparison test for post hoc analysis using SPSS 19.0 (SPSS Inc., Chicago, IL, USA). A threshold *p*-value of < 0.05 was adopted to indicate statistical significance. GraphPad Prism version 10.0 (GraphPad Software Inc., San Diego, CA, USA) was used for figure preparation.

## 3. Results

### 3.1. Comparison of Nutritional Components Between Se-DM and DM

The nutritional elemental composition of Se-DM and regular donkey milk was analyzed using ICP–MS. As shown in Table 1, Se-DM contained higher concentrations of trace elements, including zinc (Zn), calcium (Ca), phosphorus (P), and Se than regular donkey milk, with a significant 241.67% increase in Se content. Additionally, the contents of vitamin C, taurine, and total protein were also high in Se-DM.

Table S3 shows the common amino acid profiles of both milk types, revealing significant increases in glutamic acid, proline, and leucine in Se-DM.

### 3.2. Regulatory Effect of Se-DM on HFD-Induced Rats

**Figure 1:**
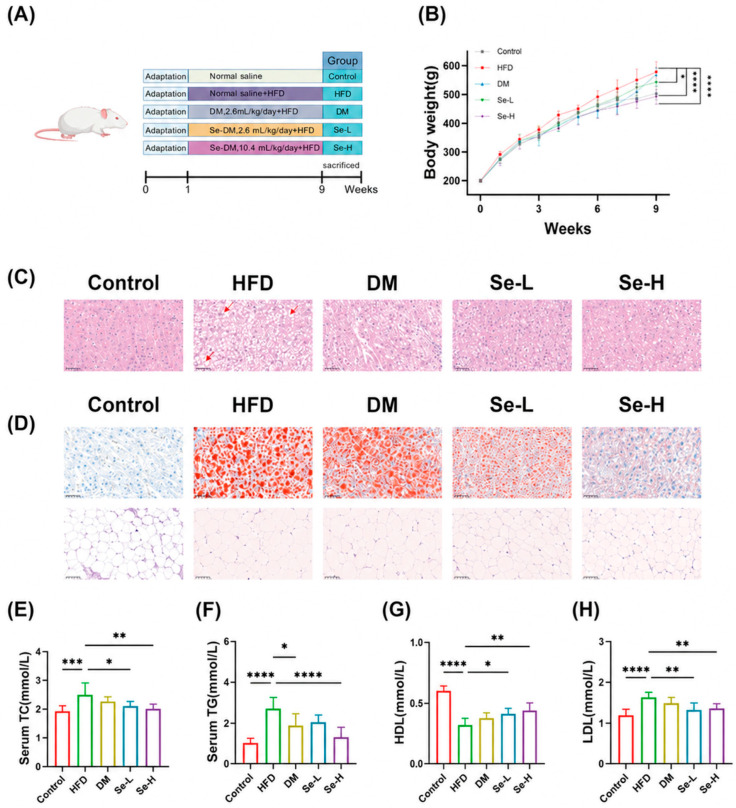
Effect of selenium-enriched donkey milk on elevated blood lipids in HFD-induced rats. (**A**) Experimental flowchart. (**B**) Body weight changes in rats. (**C**) H&E staining of rat liver tissue sections. The red arrows indicate hepatocyte degeneration and cytoplasmic vacuolization in high-fat diet-fed rats. (**D**) Oil Red O staining of rat liver tissue sections and epididymal adipose tissue sections. (**E**) Serum cholesterol level. (**F**) Serum triglyceride level. (**G**) Serum HDL-C level. (**H**) Serum LDL-C level. Control, normal diet; HFD, high-fat diet; Se L, low-dose of Se-DM intervention; Se-H, high-dose of Se-DM intervention; and DM, normal donkey milk intervention. Data are expressed as mean ± SD (n = 8). * *p* < 0.05, ** *p* < 0.01, *** *p* < 0.005, and **** *p* < 0.001. Scale bar, 50 μm.

**Figure 2:**
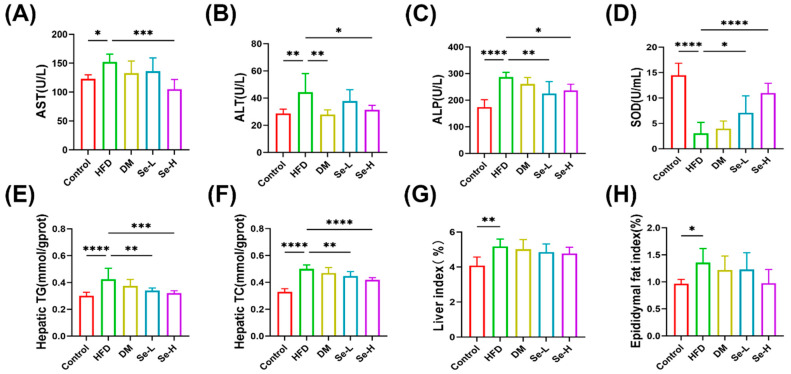
Effect of selenium-enriched donkey milk on lipid accumulation in HFD-induced rats. (**A**) Serum AST level. (**B**) Serum ALT level. (**C**) Serum ALP level. (**D**) Serum SOD level. (**E**) Hepatic triglyceride level. (**F**) Hepatic cholesterol level. (**G**) Liver index. (**H**) Epididymal fat index. Data are expressed as mean ± SD (n = 8). * *p* < 0.05, ** *p* < 0.01, *** *p* < 0.005, and **** *p* < 0.001.

The overall flowchart of the experiment is shown in Figure 1A. We evaluated the preventive effects of Se-DM on lipid accumulation in HFD-fed rats by analyzing lipid profiles, body weight, as well as liver and epididymal fat tissue sections. The results showed that body weight gain in rats was effectively controlled after Se-DM administration (Figure 1B). H&E staining results showed that Se-DM partially prevented hepatocyte degeneration and cytoplasmic vacuolation in HFD-fed rats. (Figure 1C). Furthermore, Oil Red O staining demonstrated that both Se-DM and regular donkey milk inhibited lipid droplet accumulation in hepatocytes and hypertrophy of epididymal adipocytes in HFD-fed rats, and Se-DM exhibited more pronounced effects (Figure 1D). Serum lipid profiles showed that the HFD group exhibited significantly higher levels of TG, TC, and LDL than the control group, along with reduced HDL (Figure 1E–H). Additionally, HFD-fed rats displayed elevated levels of ALT, AST, SOD, and ALP. These parameters were reduced in the Se-DM group (Figure 2A–D). Rats fed a high-fat diet showed marked increases in liver TC and TG levels as well as liver and epididymal fat coefficients, whereas these parameters were reduced in the Se-DM group (Figure 2E–H). These observations indicate that Se-DM effectively prevents fat accumulation induced by a high-fat diet (HFD) and protects against adverse effects on biochemical markers associated with lipid metabolism.

### 3.3. Effect of Se-DM on Intestinal Metabolism of HFD-Induced Rats

**Figure 3:**
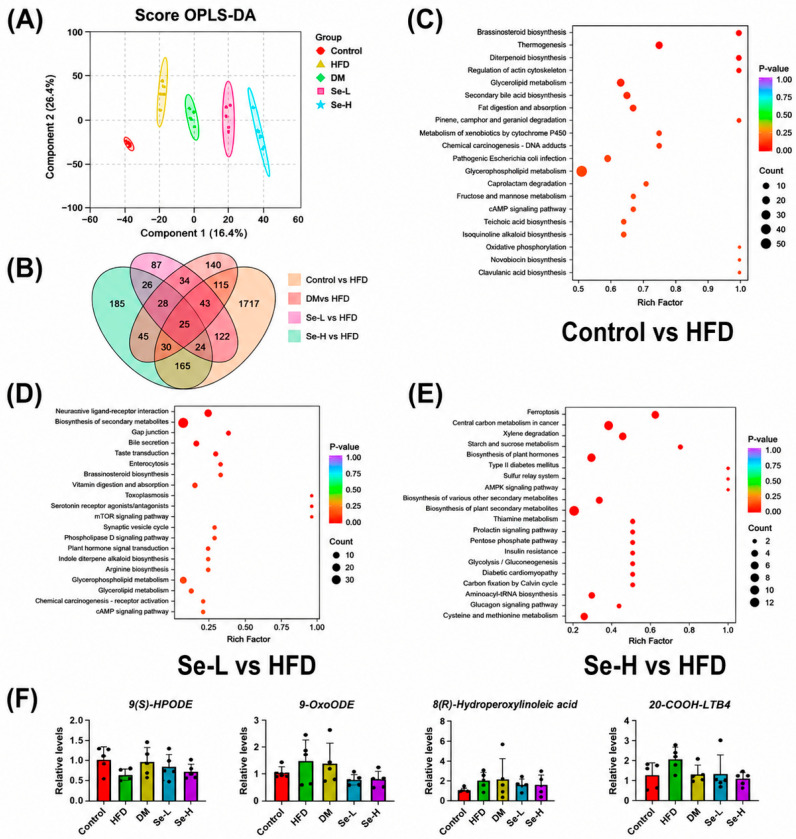
Effect of Se-DM on metabolic disorders in HFD-induced rats. (**A**) OPLS–DA score plot of metabolites. (**B**) Venn diagram of differential metabolites. (**C**) KEGG enrichment analysis of differential metabolites between the Control and HFD groups. (**D**) KEGG enrichment analysis of differential metabolites between the Se-L and HFD groups. (**E**) KEGG enrichment analysis of differential metabolites between the Se-H and HFD groups. (**F**) Relative expression levels of 9-OxoODE, 9(S)-HPODE, 8(R)-Hydroperoxylinoleic acid, and 20-COOH-LTB4. Data are expressed as mean ± SD (n = 8).

Metabolites of intestinal microbiota mediate the interaction between the host and intestinal flora. To further explore how Se-DM regulates metabolism in HFD-fed rats, we comprehensively analyzed the fecal samples from the control, HFD, DM, Se-L, and Se-H groups using UHPLC-Q-TOF MS. Orthogonal partial least squares–discriminant analysis (OPLS–DA) revealed clear separation of metabolites across the groups (Figure 3A). A Venn diagram illustrated the numbers of metabolites unique to or shared among the groups. Differential metabolite counts between the HFD and control groups, DM and HFD groups, Se-L and HFD groups, and Se-H and HFD groups were 2241, 460, 389, and 528, respectively (Figure 3B). KEGG pathway enrichment analysis was conducted to assess the relationships among differential metabolites in response to DM and Se-DM treatments (Figure 3C–E and Figure S1). Metabolites differing between the control and HFD groups were mostly related to glycolipid metabolism, fat digestion and absorption, and fructose and mannose metabolism (Figure 3C). Substantial intergroup differences were noted for the DM and HFD groups across several metabolic pathways, namely oxidative phosphorylation, glyoxylate and dicarboxylate metabolism, phenylalanine metabolism, and pantothenate and coenzyme A synthesis (Figure S1). The metabolic differences between the Se-L and HFD groups were mainly associated with the biosynthesis of secondary metabolites, the mTOR signaling pathway, and glycerophospholipid metabolism (Figure 3D). In the Se-H group, metabolites related to ferroptosis and the AMPK signaling pathway significantly changed compared with the HFD group. Moreover, pathways involved in glucose and lipid metabolism, namely type II diabetes mellitus, the pentose phosphate pathway, and insulin resistance, underwent distinct shifts (Figure 3E). Furthermore, in the HFD group, oxidized lipids derived from the linoleic acid and arachidonic acid pathways (9-OxoODE, 9(S)-HPODE, 8(R)-hydroperoxylinoleic acid, and 20-COOH-LTB4) were upregulated, but they were downregulated in the Se-H group (Figure 3F). Therefore, our results show that Se-DM intake may regulate lipid peroxidation and lipid metabolism in HFD-fed rats through the aforementioned pathways.

### 3.4. Effect of Se-DM on Intestinal Dysbiosis Induced by HFD

Maintaining intestinal homeostasis and regulating health are essential functions of intestinal microbiota. HFD is often associated with intestinal microbiota dysbiosis, and intestinal microbiota are closely associated with intestinal lipid absorption and hepatic lipid metabolism [8]. To investigate the effects of DM and Se-DM on the intestinal microbiota of HFD-fed rats, the composition of fecal microbiota in rats was analyzed using 16S rRNA sequencing.

**Figure 4:**
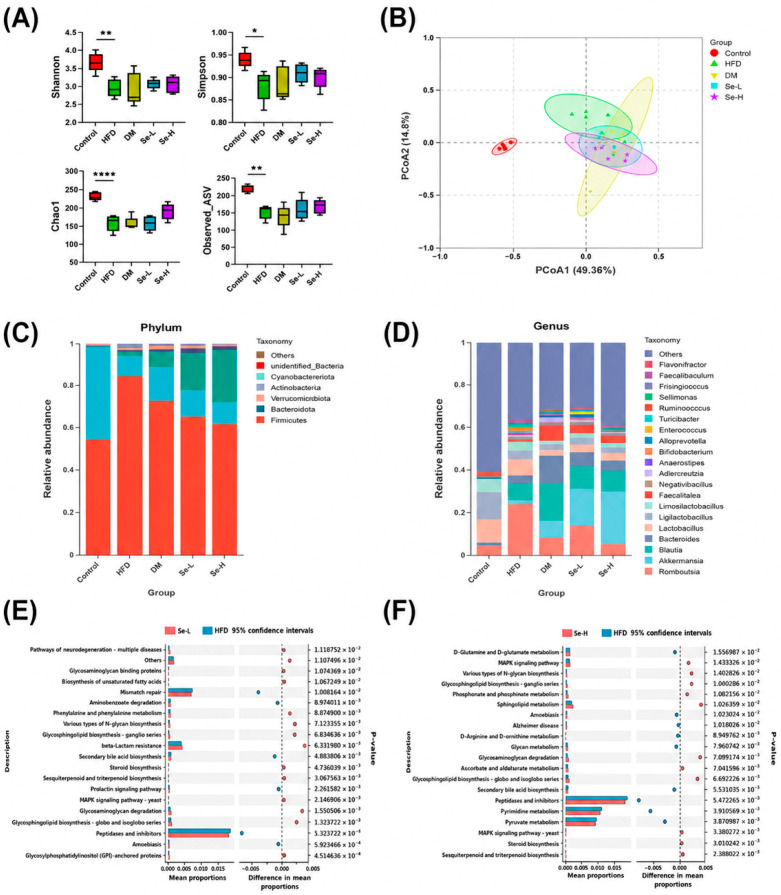
Effect of Se-DM on intestinal microbiota dysbiosis in HFD-induced rats. (**A**) α-Diversity indices of HFD-induced rats. (**B**) PCoA plot of intestinal microbiota at the ASV level. (**C**) Changes in intestinal microbiota at the phylum level. (**D**) Changes in intestinal microbiota at the genus level. (**E**) STAMP plot of KEGG enrichment between the Se-L and HFD groups. (**F**) STAMP plot of KEGG enrichment between the Se-H and HFD groups. Data are expressed as mean ± SD (n = 8). * *p* < 0.05, ** *p* < 0.01 and **** *p* < 0.001.

α-Diversity index results (Sobs, Shannon, Simpson, and Chao1) showed that HFD feeding significantly reduced the diversity and abundance of intestinal microbiota relative to the control group. Despite the lack of a statistically significant difference, the Se-H group numerically restored these indices (Figure 4A). According to principal coordinate analysis (PCoA) based on ASV abundance, intestinal microbiota composition exhibited distinct responses to HFD and high-dose Se-DM (Figure 4B).

KEGG pathway analysis was performed on the rat fecal microbiota (Figure 4E,F, and Figure S2). Downregulation of the “arachidonic acid metabolism” and “alcoholism” pathways was observed in the DM group versus the HFD group, as is shown by Level 3 data (Figure S3), the Se-L group exhibited downregulation of the “beta-lactam resistance” pathway (Figure 4E), and the Se-H group exhibited increased activity of the “AMPK signaling pathway” and “sphingolipid metabolism” pathway (Figure 4F).

**Figure 5:**
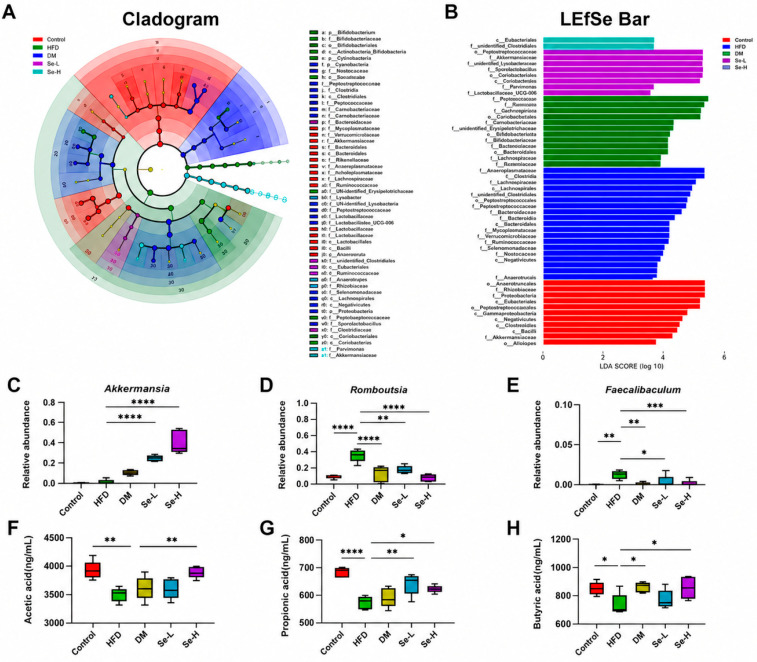
Effect of Se-DM on intestinal homeostasis and SCFA production in HFD-induced rats. (**A**,**B**) Linear discriminant analysis effect size (LEfSe) and LDA scores of intestinal microbiota. (**C**) Relative abundance of Akkermansia. (**D**) Relative abundance of Romboutsia. (**E**) Relative abundance of Faecalibaculum. (**F**) Acetate content in feces. (**G**) Propionate content in feces. (**H**) Butyrate content in feces. Data are expressed as mean ± SD (n = 8). * *p* < 0.05, ** *p* < 0.01, *** *p* < 0.005, and **** *p* < 0.001.

Firmicutes and Bacteroidota constituted the dominant phyla in rat feces. HFD feeding led to an overabundance of Firmicutes and a decrease in Bacteroidota; DM and Se-DM supplementation alleviated this condition and enhanced the relative abundance of Verrucomicrobiota (Figure 4C). At the genus level, abundances of Faecalibaculum and Romboutsia (genera that exacerbate fat accumulation) in the HFD group increased. Se-DM treatment mitigated the above changes and significantly increased the abundance of Akkermansia (Figure 4D and Figure 5C–E).

A linear discriminant analysis effect size (LEfSe) was used to characterize the representative intestinal microbiota of rats, and linear discriminant analysis (LDA) was employed to detect significant differences in intestinal microbiota across the four experimental groups (Figure 5A,B). The results revealed that Se-DM ameliorated HFD-induced intestinal dysbiosis and facilitated the proliferation of *A. muciniphila*. *A. muciniphila* is a well-known probiotic with multiple functions, such as reducing fat accumulation, enhancing intestinal immunity, and promoting the production of SCFAs. Overall, our findings demonstrate that after treatment with selenium-enriched donkey milk, the intestinal abundance of *A. muciniphila* in rats was significantly increased, and the gut microbial homeostasis was restored.

### 3.5. Effect of Se-DM on Production of SCFAs

Gut microbiota ferment dietary fiber to produce short-chain fatty acids (SCFAs), which are key mediators of digestion, immune function, and neurological processes. Additionally, they significantly contribute to gut homeostasis, fat absorption, and hepatic lipid metabolism. SCFA contents in rat feces were quantified using GC–MS. The results showed that DM pretreatment moderately increased butyrate content. Relative to the HFD group, propionate content exhibited partial normalization in the Se-L group, while the Se-H group substantially attenuated the HFD-associated decline in total SCFAs—namely acetate, propionate, and butyrate (Figure 5F–H and Figure S3). These results suggested that Se-DM, potentially through increased SCFA production, prevented hepatic lipid metabolism disorder and regulated lipid peroxidation in rats.

### 3.6. Effects of Se-DM on Ferroptosis and Lipid Peroxidation Induced by HFD and De Novo Fatty Acid Synthesis by Activating the AMPK Pathway

**Figure 6:**
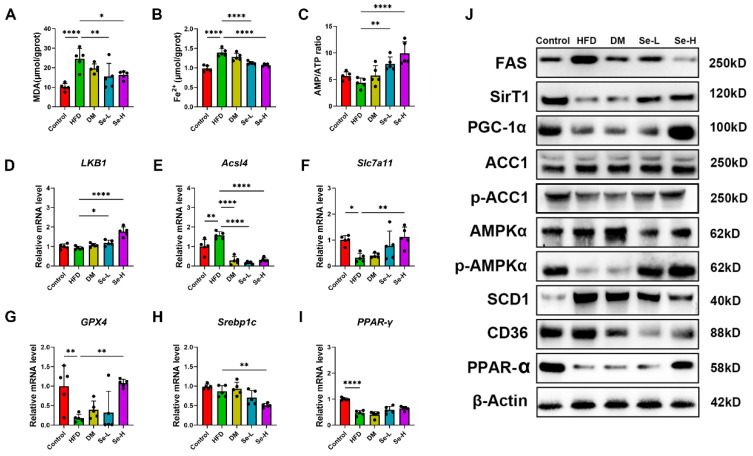
Effect of Se-DM on the expression of genes and proteins related to lipid metabolism disorders in HFD-induced rats. (**A**) MDA level in liver tissue. (**B**) Ferrous ion level in liver tissue. (**C**) AMP/ATP ratio in liver tissue. (**D**) Relative mRNA level of LKB1. (**E**) Relative mRNA level of ACSL4. (**F**) Relative mRNA level of Slc7a11. (**G**) Relative mRNA level of GPX4. (**H**) Relative mRNA level of Srebp1c. (**I**) Relative mRNA level of PPAR-γ. (**J**) Expression of lipid metabolism-related proteins in liver tissue. Data are expressed as mean ± SD (n = 8). * *p* < 0.05, ** *p* < 0.01 and **** *p* < 0.001.

To explore markers associated with the AMPK pathway and ferroptosis in rats, we assessed the levels of Malondialdehyde (MDA), ferrous ions, and glutathione (GSH). Additionally, the AMP/ATP ratio in rat livers was measured. Moreover, we performed Western blot analysis and qPCR on rat liver tissues (Figure 6A–J).

We observed reduced hepatic MDA and ferrous ion levels after Se-DM treatment (Figure 6A,B), while the GSH content and AMP/ATP ratio increased (Figure S4 and Figure 6C). qPCR results revealed (Figure 6D–I) that Se-DM intervention downregulated the ferroptosis-promoting gene ACSL4 while upregulating the ferroptosis-inhibiting genes LKB1, GPX4, and Slc7a11. In parallel, the expression of Srebp1c and PPAR-γ, which are involved in fatty acid synthesis, was elevated following Se-DM supplementation.

As shown in Figure 6J, Se-DM treatment partially prevented the disruption of lipid metabolism and ferroptosis attributed to HFD. Notably, the protein levels of FASN, SCD1, and the levels of AMPKα, p-AMPKα, SirT1, PGC-1α, and PPAR-α (involved in ferroptosis and lipid peroxidation) were restored.

The above results reveal that Se-DM affects ferroptosis-mediated lipid peroxidation by inhibiting fatty acid synthesis and stimulating the AMPK pathway, thereby eliminating the dysregulation of lipid metabolism induced by an HFD.

### 3.7. Regulation of SCFAs on Lipid Peroxidation in HepG2 Cells

**Figure 7:**
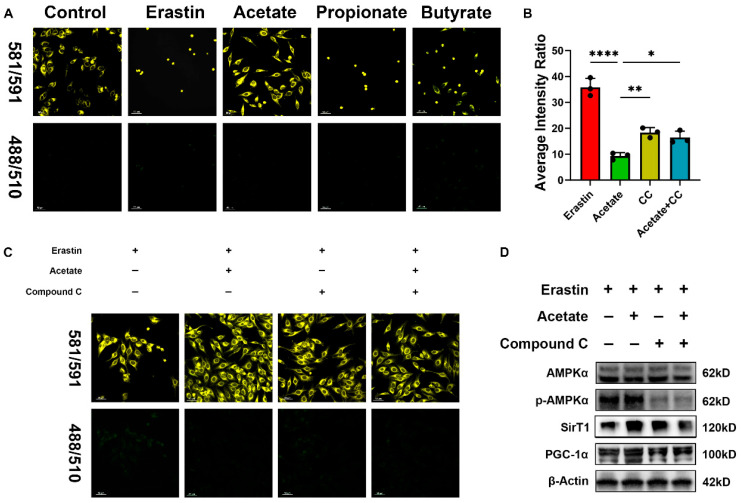
Effect of SCFAs on lipid peroxidation and mitochondrial metabolism in HepG2 cells. (**A**) Acetate, propionate, and butyrate-treated HepG2 cells. (**B**,**C**) Imaging and relative fluorescence quantification of HepG2 cells treated with acetate. (**D**) Protein expression in HepG2 cells. Dye: C11 BODIPY. Average intensity ratio = [Average intensity (488/510)/Average intensity (581/591)] × 100. ImageJ software was used for quantification. Data are expressed as mean ± SD (n = 3). * *p* < 0.05, ** *p* < 0.01 and **** *p* < 0.001.

To determine whether SCFAs act as activators of the AMPK pathway, we conducted experiments using the ferroptosis inducer erastin and the AMPK inhibitor Compound C. Then, the lipid peroxidation was assessed using the C11-BODIPY probe and visualized by a high-resolution laser confocal microscope. The results showed that acetate significantly mitigated erastin-induced cell death and lipid peroxidation levels (444/510 nm) compared with propionate and butyrate (Figure 7A). However, this protective effect was abolished in the presence of Compound C (Figure 7B,C). Western blot analysis further revealed changes in the expression of relevant proteins (Figure 7D). These findings show that SCFAs, particularly acetate, serve as critical mediators in activating the AMPK pathway.

### 3.8. Promotional Effect of the Functional Components of Se-DM on the Proliferation of A. muciniphila

**Figure 8:**
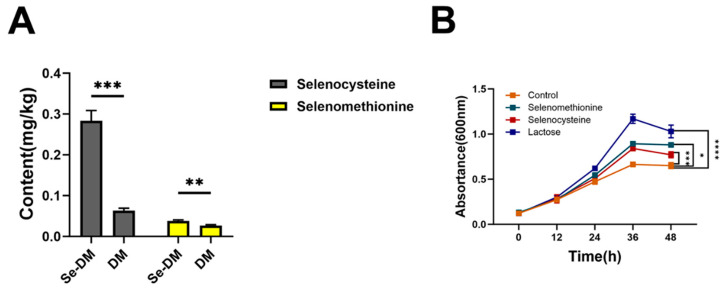
Composition of organic Se in Se-DM and DM, and the effect of organic Se on the growth of Akkermansia muciniphila. (**A**) Forms of organic Se in DM and Se-DM. (**B**) Effects of adding SeCys, SeMet, and lactose on the growth of *A. muciniphila*. Data are expressed as mean ± SD (n = 3). * *p* < 0.05, ** *p* < 0.01, *** *p* < 0.005, and **** *p* < 0.001.

To identify the functional components in Se-DM that increase the abundance of *A. muciniphila*, we determined the forms of organic Se in Se-DM using HPLC-ICP-MS. The results showed that SeCys and SeMet were identified as the major components of organic Se, while other Se components were not detected (Figure 8A).

Studies have shown that human milk oligosaccharides are beneficial for the growth of *A. muciniphila* [17]. As a potent alternative to human milk, the effects of functional components in Se-DM on *A. muciniphila* have not been fully explored. We added SeCys, SeMet, and lactose to culture dishes and measured the growth curve. The results showed that SeCys and SeMet promoted the proliferation of *A. muciniphila* (Figure 8B).

## 4. Discussion

HFD serves as a primary driver of obesity. It also induces metabolic disturbances characterized by elevated TC, TG, and LDL-C alongside reduced HDL-C levels. HFD can lead to ectopic lipid deposition and excessive accumulation, thereby causing chronic inflammation. Clinicians frequently assess serum AST and ALT to evaluate liver damage [1,18]. In this study, Se-DM reduced the levels of TC, TG, LDL-C, AST, and ALT but increased the level of HDL-C. Additionally, histological analysis revealed that Se-DM reduced hepatic lipid accumulation, cellular degeneration, and epididymal fat enlargement. We confirmed that Se-DM increased the serum SOD content and decreased the MDA and reactive oxygen species (ROS) contents in HFD-induced rats. SOD, ROS, and MDA reflect the levels of lipid peroxidation and oxidative stress caused by lipid accumulation. These results showed that Se-DM eliminated the symptoms of HFD-induced rats by mitigating lipid peroxidation and reducing oxidative stress.

HFD significantly altered cellular energy regulation and metabolic homeostasis and affected the mRNA and protein expression levels of the target gene (FASN, SCD1, AMPKα, p-AMPKα, SirT1, PGC-1α, PPAR-α, etc.). The untargeted metabolomics and 16S microbiota analysis revealed that Se-DM intervention upregulated AMPK-related metabolites and downregulated ferroptosis-related metabolites. This change was further confirmed through the determination of ferrous ion levels and the AMP/ATP ratio in the liver. AMPKα is a key regulatory target for energy homeostasis and is closely associated with the occurrence of ferroptosis [19]. Ca^2+^/calmodulin-dependent kinase kinases (CaMKKs) and liver kinase B1 (LKB1) were the upstream genes of AMPK, which activated AMPKα through phosphorylation at Thr172. AMPKα activation led to reduced expression of acetyl-CoA carboxylase 1 (ACC1) and enhanced its phosphorylation. ACC1 serves as a critical rate-limiting enzyme in fatty acid synthesis, modulating the production of various polyunsaturated fatty acids (PUFAs), including arachidonic acid and adrenic acid [15,16]. In addition, inhibition of ACC1 suppressed the expression of the downstream sterol regulatory element-binding protein 1c (Srebp1c), fatty acid synthase (FASN), and stearoyl-CoA desaturase 1 (SCD1), thereby inhibiting weight gain and hepatic steatosis [20]. Peroxisome proliferator-activated receptor-γ coactivator 1α (PGC-1α) and deacetylase Sirtuin 1 (SIRT1) play crucial roles in mitochondrial energy metabolism and act as downstream targets of AMPK [21,22]. Experimental results showed that Se-DM increased the expression levels of LKB1, phosphorylated AMPKα (p-AMPKα), phosphorylated ACC1 (p-ACC1), PGC-1α, and SIRT1 but inhibited the expression of Srebp1c, FASN, and SCD1. In addition, the scavenger receptor CD36 (an upstream component of AMPK signaling) was downregulated, whereas peroxisome proliferator-activated receptors PPAR-α and PPAR-γ showed elevated expression. These findings reveal that Se-DM administration reduces lipid transport and fatty acid production, increases cellular energy metabolism, and restores hepatic lipid homeostasis in rats by activating the phosphorylation of AMPK.

Solute carrier family 7 member 11 (Slc7a11) and GSH-dependent phospholipid hydroperoxidase (GPX4) are inhibitors of ferroptosis. Slc7a11 accelerates the utilization of cystine for synthesizing reduced GSH, which acts as a cofactor for GPX4 in reducing lipid peroxides and suppressing ferroptosis [23,24]. HFD-induced rats exhibited overexpression of long-chain acyl-CoA synthetase 4 (ACSL4). ACSL4 preferentially catalyzed arachidonic acid, esterifying it into phospholipids containing oxidized PUFAs, thereby triggering ferroptosis. The results showed that Se-enriched donkey milk (Se-DM) treatment significantly attenuated the increase in ACSL4, upregulated Slc7a11 and GPX4, and reduced the content of pro-inflammatory oxidized lipids in metabolites (Figure S5), thereby inhibiting ferroptosis.

SCFAs are microbial fermentation products in the gut, mainly comprising acetate, propionate, and butyrate. G protein-coupled receptors (GPR41 and GPR43) in intestinal cells mediate the absorption of SCFAs, thereby accelerating insulin secretion and improving glucose metabolism. The gut microbiota of the host is modulated by various environmental factors, among which differences in the amount and type of individual dietary intake play key roles. Dietary patterns have been reported to remodel the gut microbiota and boost SCFA production, thereby regulating human metabolism through multiple pathways [8,25]. The gut–liver axis serves as a key pathway through which SCFAs exert their biological effects.

The biliary tract, portal vein, and systemic circulation serve as the anatomical connections between the liver and intestines. Venous blood flows from the intestines through the portal vein into the hepatic sinusoids for detoxification, then enters the hepatic veins, and finally returns to the heart and lungs. During this process, bioactive substances such as bile acids released by the liver enter the intestines. Intestinal microbiota and the host together decompose endogenous and exogenous substrates, and the products (e.g., SCFAs and secondary bile acids) flow back to the liver through the bloodstream [26]. *A. muciniphila* colonizes the intestinal mucosal layer and imparts multiple benefits, including SCFA production, improved intestinal integrity, and inhibition of lipopolysaccharide (LPS) translocation from the intestines [27,28]. Recent studies have shown that *A. muciniphila* can eliminate various symptoms induced by HFD feeding, including weight reduction, relief of insulin resistance, and improved fasting hyperglycemia. We analyzed the fecal microbiome composition and determined SCFA contents. Se-DM intervention led to a significant increase in the abundance of *A. muciniphila* and elevated levels of SCFAs (acetate, propionate, and butyrate) [29]. The impact of SCFAs on ferroptosis was subsequently validated using a HepG2 cell model. Therefore, Se-DM can alleviate hepatic lipid accumulation and lipid peroxidation through the gut–liver axis by promoting SCFA production through increasing *A. muciniphila* abundance, and this beneficial effect may be attributed to AMPK pathway activation by acetate [30,31].

Increasing the Se content in dairy products is a common nutritional fortification strategy. Feeding lactating animals with Se-enriched feed and fermenting milk using Se-enriched microorganisms are common approaches to obtain Se-enriched dairy products. Se in milk mainly exists in the form of organic Se, including SeMet, MeSeCys, and SeCys, which are crucial for the synthesis of GPX4 [8,10,13,14]. Studies have shown that Se compounds, such as MeSeCys, SeCys, SeMet, and sodium selenite, can all reduce erastin-induced ferroptosis in vitro [32]. In addition, SeCys contributes to the control of inflammation and the maintenance of redox homeostasis. SeMet can alleviate atherosclerosis and cognitive impairment, and MeSeCys can improve mitochondrial metabolism [33,34,35,36]. We analyzed the composition and content of organic Se in Se-DM using HPLC-ICP-MS and added SeCys, SeMet, and lactose to the in vitro culture of *A. muciniphila* [37,38]. The detection results showed that selenocystine and SeMet were the main organic Se components in Se-DM. Findings from in vitro cultures indicated that although selenocystine and selenomethionine (SeMet) alone exhibited weaker stimulatory effects on Akkermansia muciniphila compared with lactose, they still increased the colony-forming unit count of this bacterium to a certain extent [39]. Notably, the lactose level in Se-enriched donkey milk (Se-DM) was increased by 16.72% compared with ordinary donkey milk, suggesting a synergistic effect between the milk matrix and selenium compounds. This synergistic effect makes Se-DM more effective than selenium supplementation alone in promoting the proliferation of *A. muciniphila*. Given the ability of selenium compounds to alleviate ferroptosis symptoms, we believe that the regulatory effect of Se-DM on lipid metabolism in HFD-induced rats is partially attributed to the synergistic proliferation-promoting effect of its functional components (including both selenium compounds and lactose) on *A. muciniphila*.

In conclusion, this study established a rat obesity model using an HFD to explore the relationship between intestinal microbiota and lipid metabolism disorders. We found that Se-DM exerted beneficial effects, effectively preventing dyslipidemia, steatosis, hepatic inflammation, and oxidative stress. Meanwhile, Se-DM inhibited SREBP-1c-mediated fatty acid synthesis and activated the AMPK pathway, thereby affecting lipid-lowering effects. This effect may be attributed to the beneficial influence of Se-DM on gut microbiota composition. Se-DM reduced the abundance of HFD-associated potentially harmful bacteria (e.g., Faecalibaculum and Romboutsia) and promoted the growth of intestinal *A. muciniphila*. *A. muciniphila* increased SCFA production, activated the AMPK pathway, and alleviated ferroptosis, thereby reducing fatty acid synthesis and restoring dysregulated lipid metabolism. These findings reveal that Se-DM may serve as a promising dietary intervention for obesity.

## 5. Conclusions

In summary, the preventive lipid-lowering and hepatoprotective effects of Se-DM can be partially attributed to its increase in *A. muciniphila* and enhancement of SCFA production, thereby alleviating oxidative stress, inhibiting fatty acid synthesis, and restoring lipid homeostasis. The AMPK/SIRT1/PGC-1α axis and the *A. muciniphila*-mediated gut–liver axis may represent novel mechanistic pathways through which Se-DM prevents NAFLD and obesity. According to these findings, Se-DM may serve as a viable dietary intervention for hyperlipidemia.

## Figures and Tables

**Table 1:** Nutritional elemental composition of selenium-enriched donkey milk and donkey milk.

Materials (mg/kg)	DM	Se-DM	Increased Rate (%)
Zn	26.7 ± 3.12	28.4 ± 2.13	6.37%
Ca	8454 ± 18.62	9064 ± 44.18 ***	7.22%
P	5845 ± 43.05	6053 ± 71.23 ***	3.56%
Se	0.024 ± 0.003	0.082 ± 0.021 **	241.67%
Vc	0.38 ± 0.07	0.42 ± 0.11	10.53%
Protein (%)	18.18 ± 3.21	19.22 ± 2.26	5.72%
Taurine	1.06 ± 0.33	1.11 ± 0.21	4.72%
Lactose	659.04 ± 22.15	769.27 ± 24.77 **	16.72%

Data are expressed as mean ± SD (n = 3). ** *p* < 0.01, *** *p* < 0.005.

## Data Availability

The original contributions presented in this study are included in the article/Supplementary Material. Further inquiries can be directed to the corresponding author.

## Supplementary Materials

The following supporting information can be downloaded at: https://www.mdpi.com/article/10.3390/foods15101640/s1, Figure S1: KEGG enrichment analysis of differential metabolites between the DM and HFD groups. Figure S2: STAMP plot of KEGG enrichment between the Control and HFD groups. STAMP plot of KEGG enrichment between the DM and HFD groups. Figure S3: Total SCFA content in feces. Figure S4: GSH content in liver. Figure S5: The changes in derived lipids within the “arachidonic acid” metabolic pathway in the KEGG enrichment analysis. Table S1: Primer sequences for RT-qPCR. Table S2: List of antibodies. Table S3: Common amino acid of DM and Se-DM.

## Abbreviations

The following abbreviations are used in this manuscript:

AMPK	AMP-activated protein kinase
HFD	High-fat diet
ICP–MS	Inductively coupled plasma mass spectrometry
SCFA	Short-chain fatty acids
Se-DM	Selenium-rich donkey milk
DM	Donkey milk
*A. muciniphila*	*Akkermansia muciniphila*
TG	Total triglyceride
TC	Total cholesterol
LDL-C	Lipoprotein cholesterol
AST	Aspartate aminotransferase
ALT	Alanine aminotransferase
ALP	Alkaline phosphatase
MDA	Malondialdehyde
SOD	Superoxide dismutase
GPX4	GSH-dependent phospholipid hydroperoxidase
