# Supplementary material for: Effect of Selenium-Enriched Donkey Milk on Lipid Metabolism Disorders Induced by a High-Fat Diet
Source: Foods. 2026 May 8;15(10):1640. doi: 10.3390/foods15101640 (PMC13206217; doi:10.3390/foods15101640)
Supplement: Supplementary file 1 [file foods-15-01640-s001.zip › foods-4246675-supplementary.pdf]

1 Tables and Figures

Table S1 Primer sequences for RT-qPCR

Name of primer	Sequences (5'to 3')
Acsl4-F	AAGAAAGGCTATGACGCCCC
Acsl4-R	GGCCACCGATCACAATCTCA
Slc7a11-F	AGGGCATACTCCAGAACACG
Slc7a11-R	GGACCAAAGACCTCCAGAATG
LKB1-F	CTTTGAGAACATCGGGAGAGG
LKB1-R	CTGTGCTGTCTAATCTGTCGG
Gpx4-F	TCCACCGTGTATGCCTTCTCC
Gpx4-R	CCTGCTGTATCTGCGCACTGGA
SREBP-1C-F	GCTGTTGGCATCCTGCTATC
SREBP-1C-R	TAGCTGGAAGTGACGGTGGT

Table S2 List of antibodies

Antibodies	Source	Identifier
ACC1	Cell Signaling Technology	#3676
p-ACC1	Cell Signaling Technology	#11818
FASN	Proteintech Group	10624-2-AP
SirT1	Proteintech Group	13161-1-AP
PGC-1α	Proteintech Group	66369-1-Ig
AMPK-α	Proteintech Group	10929-2-AP
p-AMPK-α	Proteintech Group	80209-6-RR
SCD1	Proteintech Group	28678-1-AP
CD36	Proteintech Group	18836-1-AP
PPAR-γ	Proteintech Group	16643-1-AP
β-Actin	Proteintech Group	20536-1-AP
HRP-Goat Anti-Rabbit Secondary Antibody	Proteintech Group	RGAR001

Table S3 Common amino acid of DM and Se-DM

Materials(g/100g)	DM	Se-DM
Aspartate	1.21±0.14	1.36±0.08
Threonine	0.52±0.02	0.59±0.05
Serine	0.62±0.12	0.71±0.06
Glutamate	2.50±0.24	2.72±0.33
Proline	1.12±0.14	1.25±0.06
Glycine	0.24±0.03	0.26±0.04
Alanine	0.46±0.11	0.50±0.09
Valine	0.75±0.10	0.82±0.11
Methionine	0.40±0.03	0.49±0.06
Isoleucine	0.58±0.23	0.62±0.15
Leucine	1.06±0.34	1.25±0.18
Tyrosine	0.54±0.04	0.52±0.04
Phenylalanine	0.55±0.05	0.61±0.05
Lysine	0.95±0.13	1.09±0.15
Histidine	0.44±0.04	0.50±0.06
Arginine	0.60±0.17	0.69±0.15

Notes: mean±SD(n=3)

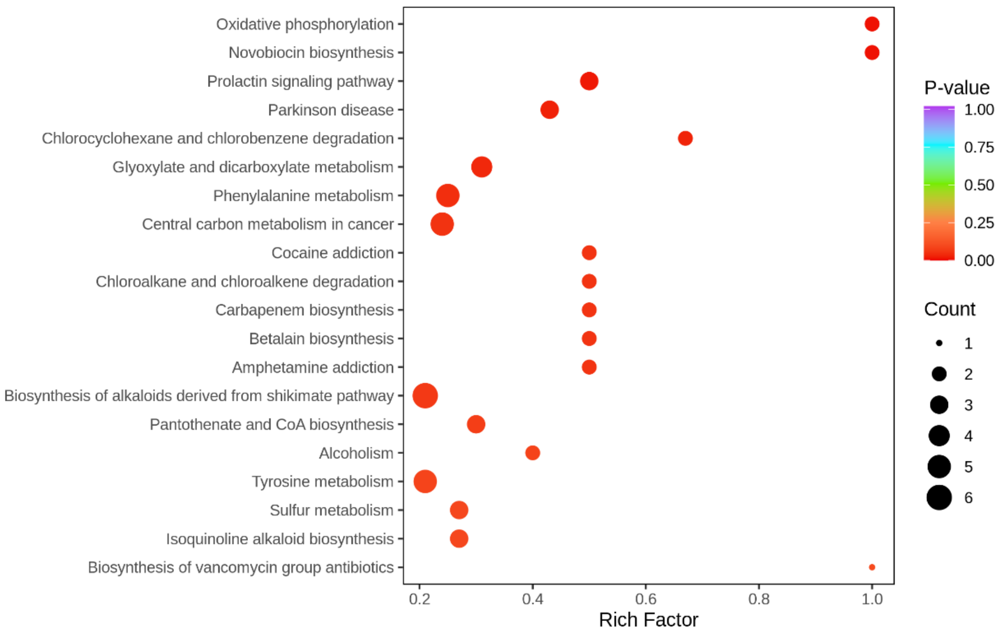

Figure S1. KEGG enrichment analysis of differential metabolites between the DM and HFD groups. Data are expressed as mean ± SD (n = 8). *p < 0.05, **p < 0.01, ***p < 0.005 and ****p < 0.001.

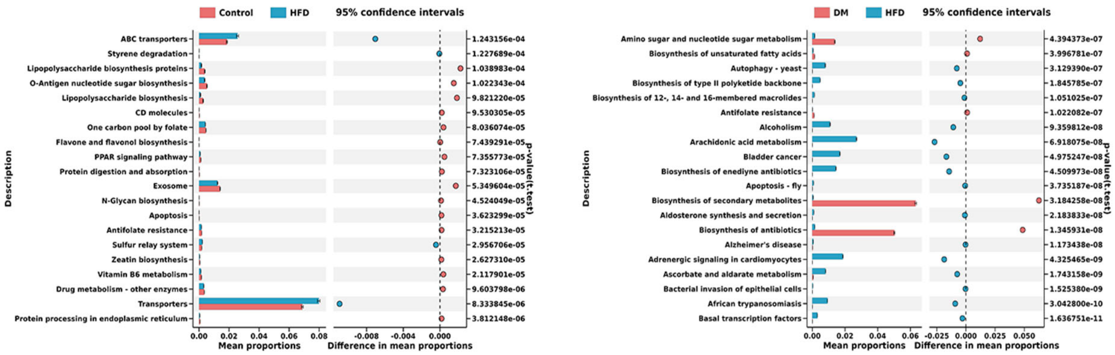

Figure S2. STAMP plot of KEGG enrichment between the Control and HFD groups. STAMP plot of KEGG enrichment between the DM and HFD groups. Data are expressed as mean ± SD (n = 8). *p < 0.05, **p < 0.01, ***p < 0.005 and ****p < 0.001.

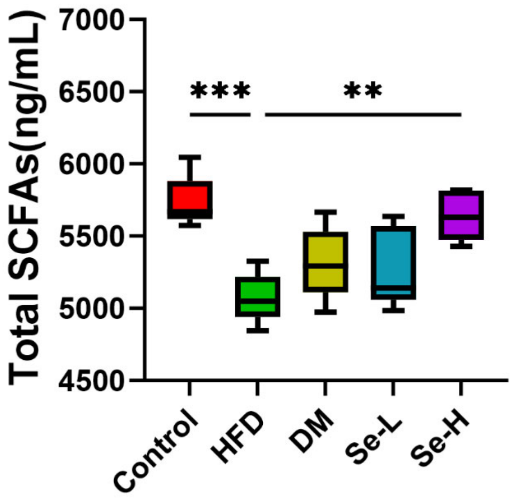

Figure S3. Total SCFA content in feces. Data are expressed as mean ± SD (n = 8). *p < 0.05, **p < 0.01, ***p < 0.005 and ****p < 0.001.

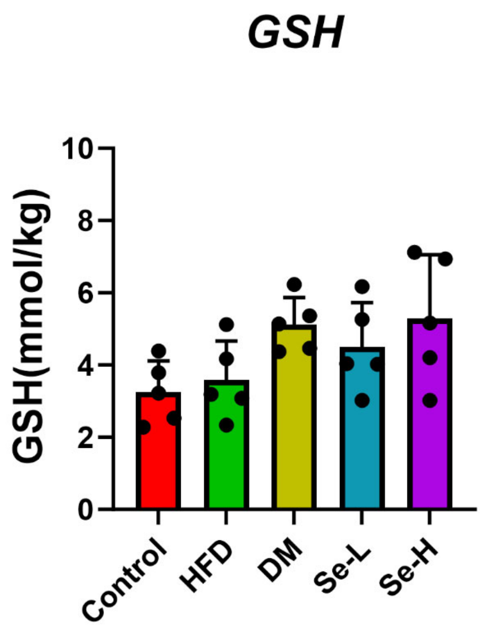

Figure S4. GSH content in liver. Data are expressed as mean ± SD (n = 8). *$p < 0.05$, **$p < 0.01$, ***$p < 0.005$ and ****$p < 0.001$.

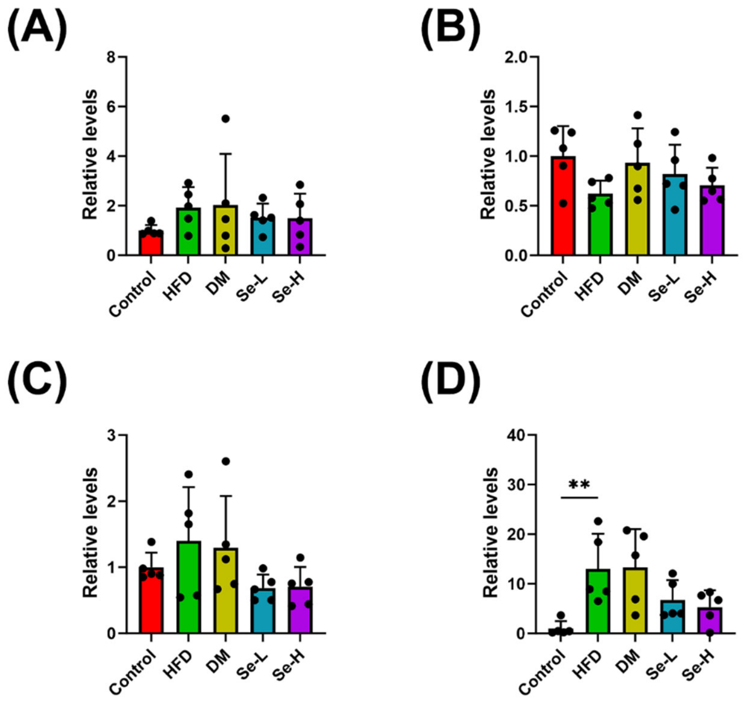

Figure S5. The changes in derived lipids within the "arachidonic acid" metabolic pathway in the KEGG enrichment analysis**.** (A) Relative expression level of 9-

OxoODE. (B) Relative expression level of 9(S)-HPODE. (C) Relative expression level of 8(R)-HPODE. (D) Relative expression level of 20-COOH-LTB4. Data are expressed as mean ± SD (n = 8). The experiment was conducted in triplicate. *$p < 0.05$, **$p < 0.01$, ***$p < 0.005$, and ****$p < 0.001$.

2 Methods

2.1 Histological analysis

Processing of liver and epididymal adipose tissues: Fresh liver and epididymal adipose tissues were rinsed with pre-chilled phosphate-buffered saline (PBS, pH 7.4) to remove residual blood. Tissues were fixed in 10% neutral buffered formalin for 48 hours, followed by gradient ethanol dehydration (70%, 80%, 90%, 95%, 100%), xylene clearing, and paraffin embedding. Serial sections of 4 μm thickness were prepared.

Hematoxylin and eosin (H&E) staining: Sections were deparaffinized and rehydrated (xylene twice, 10 minutes each; 100% ethanol, 95% ethanol, 80% ethanol, 70% ethanol for 5 minutes each; distilled water twice). Sections were stained with hematoxylin solution for 5 minutes, rinsed in running water for 15 minutes for blue development, then stained with eosin solution for 2 minutes. After gradient ethanol dehydration (70%, 80%, 90%, 95%, 100% for 1-2 minutes each) and xylene clearing, sections were mounted with neutral balsam.

Oil Red O staining: Tissues were embedded in OCT compound and sectioned at 8 μm thickness using a cryostat at –20°C. Sections were air-dried for 30 minutes at room temperature and fixed in 10% neutral buffered formalin for 10 minutes. Sections were rinsed in 60% isopropanol for 1 minute, then stained with freshly prepared Oil Red O working solution (0.5% Oil Red O in isopropanol diluted with distilled water at a 3:2 ratio) for 15 minutes in the dark. Differentiation was performed with 60% isopropanol to remove background, followed by three washes with distilled water. Nuclei were counterstained with hematoxylin for 1 minute, and sections were mounted with aqueous mounting medium.

Quantification of hepatic steatosis: ImageJ software (National Institutes of Health, USA) was used to analyze Oil Red O-stained sections from 5 animals per group, with at least 5 non-overlapping fields per animal (200× magnification). The

percentage of Oil Red O-positive area relative to the total field area was calculated, and the mean values were used as quantitative indicators of lipid deposition.

Quantification of epididymal adipocyte size: Under 200× magnification, 5 randomly selected fields per animal were examined, and the cross-sectional areas (μm^2) of at least 50 adipocytes were statistically compared between groups.

To ensure objectivity and reliability of the evaluation results, morphological assessment and quantitative analysis of histological sections were performed using a blinded method. Specifically, all sections were uniformly coded and independently evaluated by two researchers who were unaware of the experimental group assignments. The results from the two researchers were tested for consistency. In case of disagreement, a third researcher performed a review and reached a consensus. Data were assigned to the corresponding experimental groups according to the coding key only during the statistical analysis phase.

2.2 RT-qPCR analysis

Total RNA was extracted from liver tissues using TRIzol reagent (Invitrogen, USA). RNA quality was assessed by the following two methods:

Purity assessment: The A260/A280 ratio of RNA samples was measured using a NanoDrop 2000 spectrophotometer (Thermo Fisher Scientific, USA). All samples used for subsequent experiments had A260/A280 ratios between 1.8 and 2.0, indicating low contamination by proteins and phenolic compounds.

Integrity assessment: A 1 μg aliquot of total RNA was subjected to 1% agarose gel electrophoresis, and the 28S and 18S ribosomal RNA bands were visualized using a gel imaging system. All samples exhibited a 28S band intensity approximately twice that of the 18S band, with no obvious smearing or degradation, indicating good RNA integrity.

All gene-specific primers were designed and synthesized by Sangon Biotech (Shanghai, China). Primer validation included the following steps:

Primer specificity validation: Conventional PCR was performed to amplify the target gene fragments, and the PCR products were subjected to 1.5% agarose gel electrophoresis to confirm that the amplified product sizes were consistent with

expectations and that no primer dimers or non-specific amplification bands were present.

Amplification efficiency validation: qPCR was performed using a serial dilution of cDNA templates (1, 1/5, 1/25, 1/125, 1/625) to construct standard curves. The amplification efficiency (E) for each primer was calculated according to the formula: $E = (10^{(-1/slope)} - 1) \times 100\%$. All primers exhibited amplification efficiencies between 90% and 110%, with correlation coefficients (R^2) greater than 0.99, indicating good amplification efficiency.

Melting curve analysis: Melting curve analysis was performed after the completion of qPCR (65°C–95°C, 0.5°C/5 sec). All genes showed a single sharp peak in the melting curve, and no non-specific amplification or primer-dimer signals were detected.

To ensure the reliability of the qPCR data, we evaluated the expression stability of the reference gene GAPDH across different experimental groups:

Expression stability analysis: The geNorm software was used to calculate the expression stability (M value) of GAPDH across different samples. The results showed that the M value of GAPDH was 0.23, which is below the recommended threshold ($M < 0.5$), indicating highly stable expression across experimental groups.

Cycle threshold (Ct) value variation analysis: The range of Ct values for GAPDH across all samples was analyzed. The results showed that the mean Ct value of GAPDH was 21.34 ± 0.42, with a standard deviation of Ct values less than 0.5 across experimental groups, further confirming the suitability of GAPDH as a reference gene.

Validation experiment: Randomly selected samples were used to compare the relative quantification results when using GAPDH versus another commonly used reference gene, β-actin. The expression trends of the target genes normalized by both reference genes were consistent, validating the reliability of GAPDH as an internal control.

qPCR reactions were performed using SYBR® Green Premix Ex Taq™ II (TaKaRa, Japan) on a LightCycler® 480 II Real-Time PCR System (Roche,

Switzerland). The reaction mixture (20 μL) contained: 10 μL SYBR Green Premix Ex Taq II, 0.8 μL forward primer (10 μM), 0.8 μL reverse primer (10 μM), 2 μL cDNA template, and 6.4 μL RNase-free ddH_2O. The reaction program was as follows: 95°C for 30 seconds for initial denaturation; 40 cycles of 95°C for 5 seconds, 60°C for 30 seconds, and 72°C for 30 seconds; followed by melting curve analysis. Each sample was run in triplicate as technical replicates. Relative expression levels were calculated using the 2^−ΔΔCt method.
